# The influence of titanium particles on the functionality of osteocytes in bone remodeling: An In-vitro study

**DOI:** 10.1371/journal.pone.0334156

**Published:** 2025-10-31

**Authors:** Neibal Almabrok, K.G. Aghila Rani, Waad Kheder, Sausan AlKawas, A.R. Samsudin

**Affiliations:** 1 Department of Oral and Craniofacial Health Sciences, College of Dental Medicine, University of Sharjah, Sharjah, United Arab Emirates; 2 Research Institute for Medical and Health Sciences, University of Sharjah, Sharjah, United Arab Emirates; 3 Restorative & Preventive Dentistry Department, College of Dental Medicine, University of Sharjah, Sharjah, United Arab Emirates; Tokushima University, JAPAN

## Abstract

Bone remodeling is a tightly controlled process coordinated by osteocytes, which regulate both bone formation by osteoblasts and bone resorption by osteoclasts. Titanium dioxide (TiO₂) implants are widely used in orthopedic and dental prosthetic rehabilitation, with likelihood of leaching titanium particles, raising concerns about their potential impact on bone cell functions. This study aimed to investigate the influence of TiO₂ microparticles (TiO2-MPs) and nanoparticles (TiO2-NPs) on the functionality of osteocytes. MLO-Y4 cells were treated with varying concentrations of TiO2-MPs or TiO2-NPs for viability studies. Cells were treated with 100 µg/mL TiO2-MPs and TiO2-NPs for 21 days, and conditioned media (CM) was obtained for quantifying sclerostin release using ELISA. Indirect osteocyte-osteoblast co-culture was developed by treating MC3T3-E1cells with CM from cells treated with 100 µg/mL TiO2-MPs and TiO2-NPs. Indirect osteocyte-osteoclast co-culture was developed by treating RAW 264.7 cells with CM and RANKL. Osteocyte-osteoblast co-culture was assayed colometrically for Alkaline Phosphatase, RANKL and OPG using ELISA; and TNF- α, IL − 1ß, OC and *Runx2* by qPCR. Mineralization was evaluated using Alizarin and calcium quantification. Osteocyte-osteoclast co-culture was assayed for TRAP and *Cat K* expression. Viability studies demonstrated 100 µg/ml MPs and NPs as a favorable concentration. Sclerostin release was particle size and time-dependent: TiO₂-MPs group, levels measured were 31.13, 14.86, 13.7, and 23.06 pg/ml over time, indicating a pronounced and sustained release compared to the TiO₂-NPs group, showing 24.3, 10.94, 10.55, and 13.71 pg/ml. Osteocyte-osteoblast co-culture showed high RANKL (1709.88 vs 155.06 pg/ml), TNF- α (16.17 vs 1.07-fold), and IL − 1ß (2.08 vs 0.92-fold) in TiO₂-MPs. ALP (12.64 U/ml) and OPG (471.45 pg/ml) were decreased with less amount of nodules in MPs CM compared to control (ALP: 19.46 U/ml; OPG: 1065 pg/ml) and NPs CM (ALP: 17.95 U/ml; OPG: 645.46 pg/ml). Osteocyte-osteoclast co-culture showed upregulation of TRAP (25.24-fold) and *Cat K* (10-fold) in MPs CM compared to both control and NPs CM. In conclusion, TiO₂ particles disrupt osteocytes functionality through release of sclerostin and RANKL that inhibit osteoblastogenesis and promote osteoclastogenesis in *in-vitro* osteocyte-osteoblast and osteocyte-osteoclast co-cultures, with microparticles behaving more harmful than nanoparticles.

## 1. Introduction

Titanium-based implants have sought high popularity in orthopedic and dental prosthetic rehabilitation compared to other metallic biomaterials over the last three decades [1,2] owing to their favorable mechanical strength, corrosion resistance, and biocompatibility. Despite their excellent metallic properties, there is still a 5% to 10% failure rate of dental implants over a 10-year period, with contributing factors including host bone quality, microbial contamination, and mechanical stress. Recent attention has turned to the release of titanium dioxide particles from the implant surfaces which may alter the peri-implant microenvironment and contribute to implant failure [3].

Recent advances in *in vitro* and *in silico* modeling have significantly enhanced our understanding of bone remodeling, particularly in response to implant materials. These models offer controlled platforms to dissect cellular responses and simulate mechanical and biochemical cues, complementing *in vivo* studies [4–6]. Despite growing recognition of the fate of titanium dioxide (TiO₂) particle-induced bone remodeling, the literature presents conflicting findings regarding their biological effects. While some studies report that TiO₂ nanoparticles impair osteogenesis and promote inflammatory responses leading to implant loosening [7], others suggest that certain surface modifications [8] or particle size range may enhance osteoblastic activity and mineralization [9]. Previous research has shown that TiO₂ particles interact with peri-implant immune cells, triggering proinflammatory responses that may affect bone remodeling leading to poor osteointegration [10]. Beyond the influence of immune cells, more emphasis has been given to the direct effect of tribocorrosion on the bone cells themselves, such as osteoblasts and osetoclast. Its impact on osteoblasts and osteoclasts has been examined to ascertain the responses of these key bone remodeling cells when exposed to TiO₂ particles [11]. Titanium particles exposure to osteoblast *in-vitro* and *in-vivo* resulted in osteoblast apoptosis, inhibited osteogenic ability and increased lipogenesis in the mouse model, while its effects on bone-resorbing osteoclast promote osteoclastic activity [12].

In contrast, few studies have examined the impact of titanium particles on osteocytes, which comprise the most abundant cell-type in bone and are its only true “permanent” resident cell population [13]. Recent bone biology studies have uncovered osteocytes behaving not only as mechanosensory cells but also as cells that play a role in paracrine and endocrine physiological functions [14,15]. These ‘trapped cells’ are arguably the least well understood and were previously assumed to have a minimal direct role in bone remodeling activity. However, recent reports on their expression of Receptor activator of nuclear factor Kappa B ligand (RANKL) and TNF that promote osteoclastogenesis and their release of sclerostin that inhibit osteoblastogenesis may suggest their direct involvement in the failure of osseointegration following exposure to titanium dioxide particles [16]. Osteocytes have been shown to respond to wear particles from biomaterials by adopting a pro-catabolic phenotype, driving perilacunar remodeling through osteocytic osteolysis, and upregulating osteolytic, pro-osteoclastogenic, and inflammatory marker expression, underscoring their multifaceted role in periprosthetic osteolysis [17,18]. Understanding this response may reveal potential therapeutic targets to prevent implant failure. The aim of this study was to investigate the effects of titanium particles on osteocyte function, with a focus on their role in bone remodeling mechanisms *in-vitro*.

## 2. Materials and methods

### 2.1. Cell culture

MLO-Y4 murine cells were maintained in alpha MEM (minimum essential medium) (Gibco, USA) supplemented with 10% fetal bovine serum (FBS) and 1% penicillin/ streptomycin (Sigma Aldrich, UK) in a humidified chamber at 370C and 5% CO2. Murine RAW264.7 macrophage cells were grown in RPMI-1640 and MC3T3-E1 pre-osteoblast cells were grown in alpha MEM media and both cells supplemented with 10% FBS and 1% penicillin/ streptomycin (Sigma Aldrich, UK), in a humidified chamber at 37oC and 5% CO2.

### 2.2. Titanium dioxide micro and nanoparticles preparation and characterization

The Titanium dioxide microparticles (TiO2-MPs) and Titanium dioxide nanoparticles (TiO2-NPs) were characterized using methods described in our previous study [10]. Five milligrams of TiO2-MPs (< 5µm) in diameter and TiO2-NPs (<100 nm) in diameter were weighed, placed in 1.5 mL microcentrifuge tubes, and UV sterilized for one hour. One mL of sterile Dulbecco’s phosphate buffered saline (DPBS) was added to each tube to reach a final concentration of 5 mg/mL. The solutions were then sonicated using Qsonica Q125-220 Sonicator (USA) equipped with a 3.2 mm diameter microtip operated at 40% in pulse mode (50 s on and off) for up to 10 min. The particles were then suspended in complete medium to prepare a final concentration of 1 mg/ mL stock solution. The hydrodynamic diameter, polydispersity index (PDI) and effective charge of TiO2- NPs and MPs were measured with a Malvern Zetasizer Nano-ZS system (Malvern Instruments, UK). All measurements were performed in complete cell culture media at 25°C using a particle concentration of 10 μg/mL for NPs and 50 μg/mL for MPs. The particle size was also analysed using SEM (VEGA3 XM-TESCAN, Czech Republic) [10].

### 2.3. Cell viability

Cultured MLO-Y4 osteocytes in T75 cm2 flasks were washed twice with PBS, detached from the plates using 1X trypsin EDTA (Sigma-Aldrich, USA), and then centrifuged at 1,200 RPM for 5 minutes at room temperature. After centrifugation, the supernatant was discarded, and the pellet was suspended in 1 mL alpha MEM. 10 µL of the cell suspension was mixed with 10 µL of trypan blue and then resuspended thoroughly. 10 µL of the mixture was then added to a hemocytometer (Neubauer Hemocytometer, USA) for cell quantification. After determining the cell number per milliliter of medium, cells were plated in triplicates at a seeding density of 5x103 cell per well in 96-well cell culture-treated plates in a final volume of 100 µL of complete alpha MEM. After reaching confluency at 24 hours, cells were treated with 25, 50, 100, 500, 1000 µg/mL of TiO2-MPs or TiO2-NPs for 24, 48, and 72 hours. At the end of each time point, images of the treated cells at each concentration were captured using the Olympus cellSens imaging software (Olympus Life Science, Tokyo, Japan). After each time point, the treatment was discarded, and the cells were incubated for 4 hours at dark with 100 µL/ well of the XTT reagent (Cell Proliferation Kit II, Roche, United Kingdom) to determine the percent cell viability post treatment with increasing concentrations of TiO2-MPs and TiO2-NPs. After the incubation period, the optical density reflecting the number of viable cells capable of cleaving the tetrazolium salts was determined using the BioTek 800 TS microplate reader. Data were derived from three biologically independent experiments, each with three technical triplicates.

### 2.4. Sclerostin assay

MLO-Y4 osteocyte cells were seeded in 24 well plates in triplicates at 5 × 104 seeding density and incubated for 24 hours. The cells were treated with 100 µg/mL TiO2-MPs and TiO2-NPs for 1,7,14 and 21 days. The level of sclerostin was quantified using enzyme-linked immunosorbent assay (ELISA) kit (Abcam, ab213889). Assays were performed following the manufacturer’s instructions. Briefly, the culture supernatant was added to Anti-Mouse Sclerostin coated microplate along with an antibody cocktail solution. After 1-hour of incubation at 37°C, the wells were washed three times with 1X wash buffer. Following this, 100 µl of TMB development solution was added and the plates were incubated with gentle shaking for 10 min in the dark. The wells were then added with 100 µL stop solution and the relative absorbance was quantified using an ELISA plate reader (Synergy HTX Multi-Mode Reader, Biotek Instruments, USA) at 450 nm. Data were derived from three biologically independent experiments, each with three technical triplicates.

### 2.5. Osteogenic induction media preparation

To induce the osteogenic differentiation of MC3T3-E1 cells, alpha MEM incomplete media was supplemented with 10 nM of dexamethasone (D4902–100 G-Sigma Aldrich), 10 mM of ß-glycerophosphate disodium salt hydrate (G9422–100 G, Sigma Aldrich), and 50 μg/mL L-ascorbic acid (A0278–100 G, Sigma Aldrich). Media was then filtered using a 0.2 μm syringe filter, and FBS and penicillin/streptomycin were added at a final concentration of 10% and 1%, respectively.

### 2.6. MLO-Y4 Conditioned media (CM) preparation

Two types of Osteocyte conditioned media (CM) were prepared for all the co-culture experiments. The first was osteocyte-microparticles conditioned media involving MLO-Y4 cells exposed to 100 µg/mL TiO2-MPs (referred to as MPs CM) and the second was osteocyte-nanoparticles conditioned media whereby MLO-Y4 cells were exposed to 100 µg/mL TiO2-NPs (referred to as NPs CM). Every third day, the media were collected and mixed with osteogenic induction media for MC3T3-E1 differentiation studies or mixed with complete RPMI media for osteoclast differentiation studies, at a ratio of 1:1.

### 2.7. Indirect co-culture of osteocyte-osteoblast

MC3T3-E1 cells were seeded in 12 well plates in triplicates at 5 × 104 seeding density and incubated for 24 hours in complete alpha MEM. After attachment and achieving 80% confluency, cells were cultured in a 1:1 mixture of osteocyte conditioned media (MPs CM or NPs CM) and osteogenic induction media, as described above. This combined medium was replenished every three days, and the cells were maintained under osteogenic conditions for 21 days.

### 2.8. Alkaline Phosphatase expression, RANKL and OPG assay

Alkaline phosphatase activity of MC3T3-E1 cells cultured in the respective MPs CM and NPs CM were measured on day 14. In preparation for this assay, cells were collected and centrifuged at 1500 rpm for 5 min [9]. ALP activity was assayed using the ALP colorimetric kit (ab83369, Abcam, United Kingdom) following the manufacturer’s protocols and readings were taken using a microplate reader at 405 nm.

On day 21, cell culture supernatants from MC3T3-E1 cells cultured in MPs CM and NPs CM were collected for detection of the amount of receptor activator of nuclear factor kappa-B Ligand (RANKL) using the mouse TRANCE/TNFSF11/RANKL ELISA Kit (RK00149, abclonal) and osteoprotegerin ELISA kit (ab100733, Abcam, USA) following the manufacturer’s protocols. Data were derived from three biologically independent experiments, each with three technical triplicates.

### 2.9. Mineralization study by Alizarin Red S staining

MC3T3-E1 cells were seeded in 12 well plates in triplicates at 5 × 104 seeding density and incubated for 24 h and subsequently treated with MPs CM and NPs CM. All treatments were prepared in osteogenic induction media and media with treatment was replenished every three days throughout 21-days.

On day-21, the cells were fixed using 4% paraformaldehyde at room temperature for 30 min. Alizarin Red Stain (ARS) (Sigma Aldrich, USA) was prepared by dissolving 680 mg of ARS powder in 50 mL of distilled water to determine calcium deposition by osteoblast cells [19]. After incubating the cells with ARS stain for 30 min, the stained nodules were captured using the Olympus cellSens imaging software.

For calcium quantification, 800 μL of 10% acetic acid (v/v) was added to the stained cells and the plate was incubated for 30 mins at room temperature with shaking. The cells were then collected using a cell scraper, and vortexed vigorously. Cell lysates were collected and kept on a heat block at 850C for 10 min, incubated on ice for 5 min, and centrifuged at 12,000 rpm for 15 min. 200 μL of NH4OH was then added to the suspensions to neutralize the acid, and the optical density was determined using a plate reader at absorbance 405 nm using the BioTek 800 TS microplate reader. A standard calibration curve for alizarin dye was prepared for quantification of total calcium release. Data were derived from three biologically independent experiments, each with three technical triplicates.

### 2.10. Osteoclast formation

Cultured RAW264.7 murine macrophage cells in T75 cm2 flasks were washed twice with DPBS, detached from the plates using a rubber scraper, and centrifuged at 1,200 RPM for 5 minutes at room temperature. After centrifugation, the supernatant was discarded, and the pellet was suspended in 1mL complete RPMI-media and cultured in a 24-well plate at a density of 3 × 104 cells/well in the presence of RANKL (100 ng/mL).

### 2.11. Indirect co-culture of osteocyte-osteoclast

RAW 264.7 cells were seeded in triplicates in 12-well plates at a density of 5 × 104 cells per well and incubated for 24 hours. The cells were then randomly divided to the following three groups. The first group was control RAW 264.7 cells treated with 100 ng/mL RANKL for 7 days, while the second and third groups were RAW 264.7 cells treated with MPs CM and RAW 264.7 cells treated with NPs CM, both under RANKL stimulation. The cells were incubated in their respective culture conditions for 7 days with media replenishment every three days.

### 2.12. Real-time PCR analysis for bone formation and resorption markers

Cells from both osteocyte-osteoblast and osteocyte-osteoclast indirect co-culture groups were seeded separately in 6-well plates in triplicates at a seeding density of 2.5 × 105 cells/ml for 21 days with media replenishment every 3 days. The MC3T3-E1 cells were maintained under osteogenic conditions. On day 21, cells were harvested, and total RNA was extracted (RNeasy Protect Mini Kit Qiagen) following the manufacturer’s protocol. RNA was then converted into cDNA using a Quantitect Reverse Transcription kit (Qiagen, Germany). Consequently, real time PCR was performed with HOT FIREPol EvaGreen qPCR Supermix (Solis Biodyne Tartu, Estonia) using the StepOne Real time PCR machine (ThermoFisher Scientific, USA). Primers used in the reaction for amplification of genes in the osteocyte-osteoblast co-culture group were TNFα, IL1β, osteocalcin (OC), and Runt-related transcription factor (RUNX) [9]. Primers targeting Tartarate Resistant Acid Phosphatase (TRAP) and Cathepsin K (Cat K) were used in the osteocyte-osteoclast indirect co-culture group (Table 1). 18S was used as an internal control for normalization of target gene expression, and relative quantification was performed using the ΔΔCt method. Data were derived from three biologically independent experiments, each with three technical triplicates.

**Table 1:** Primer sequences for genes used in real-time PCR.

Gene	Primer Sequence (Forward)	Primer Sequence (Reverse)
18S	5’-GGAGAGGGAGCCTGAGAAAC-3’	5’-CCTCCAATGGATCCTCGTTA-3’
TNF- α	5′-CAAGGACAGCAGAGGACCAG− 3′	5′-TCCTTTCCAGGGGAGAGAGG− 3′
IL − 1ß	5′-AACCTCTTCGAGGCACAAGG− 3′	5′-AGCCATCATTTCACTGGCGA− 3′
Osteocalcin	5′- CACCGAGACACCATGAGAGC− 3′	5′- CTCTTCACTACCTCGCTGCC− 3′
Runx2	5’-ACGCGAGTCTGTGTTTTTGC- 3’	5’-CATGGTGCGGTTGTCGTG- 3’
TRAP	5’-GGCTACTTGCGGTTTCACTATG-3’	5’-GGGAGGCTGGTCTTAAAGAGTG-3’
Cathepsin K	5’-CAGCTTCCCCAAGATGTGAT-3’	5’-AGCACCAACGAGAGGAGAAA-3’

### 2.13. Statistical analysis

Statistical analysis was carried out using GraphPad Prism (version 9.1.1 USA). All quantitative data were derived from triplicate samples of three separate investigations. Data were tested for normality using the Shapiro-Wilk test, and homogeneity of variances was assessed using built-in options available in GraphPad Prism. A one-way ANOVA followed by multiple comparisons was used for experiments involving estimation of sclerostin, ALP, RANKL and quantification of calcium release. A two-way ANOVA followed by multiple comparisons was used for experiments involving cell viability and real-time PCR. All assumptions for ANOVA were verified and met. Data are given as mean ± SEM. The value of p < 0.05 was considered statistically significant.

## 3. Result

### 3.1. Osteocyte viability following treatment with TiO₂-MPs and NPs

**Fig 1:**
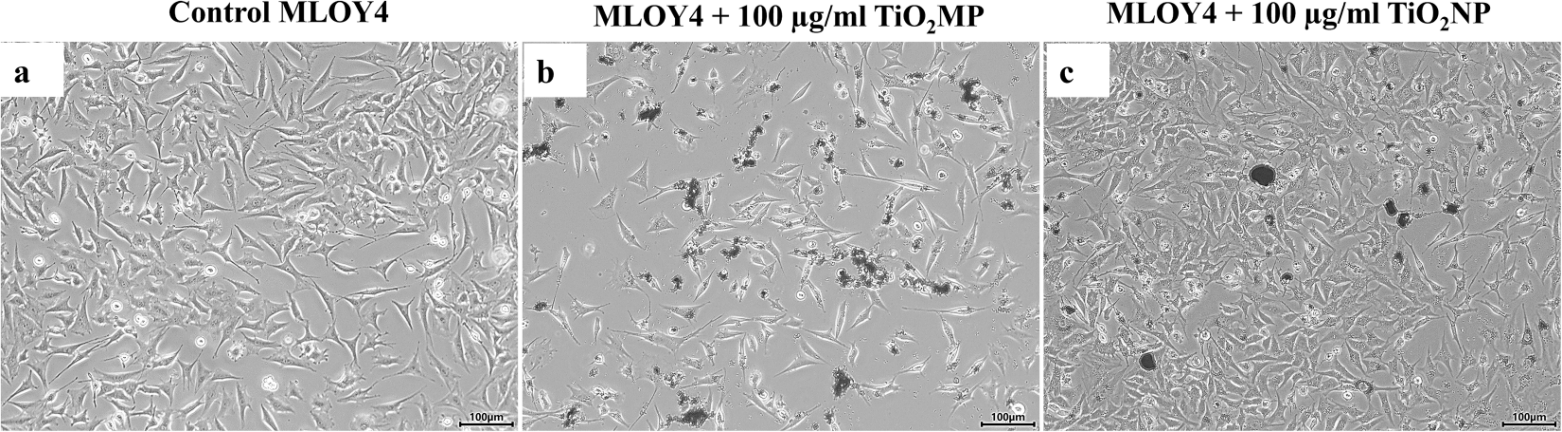
MLO-Y4 cell attachment post-treatment with TiO2-MPs and NPs along with untreated control cells. Microscopy images of **(a)** control MLO-Y4 cells, **(b)** MLO-Y4 cells treated with 100 µg/ml TiO2-MPs, and **(c)** NPs at 24h of culture. Scale bar = 100 µm for all images.

Cell viability assays demonstrated a concentration and time-dependent decline in MLO-Y4 viability upon treatment with both TiO₂-MPs and NPs. Photomicrographic images of MLO-Y4 osteocytic cells treated with TiO₂-MPs and NPs along with untreated control are shown in Fig 1.

**Fig 2:**
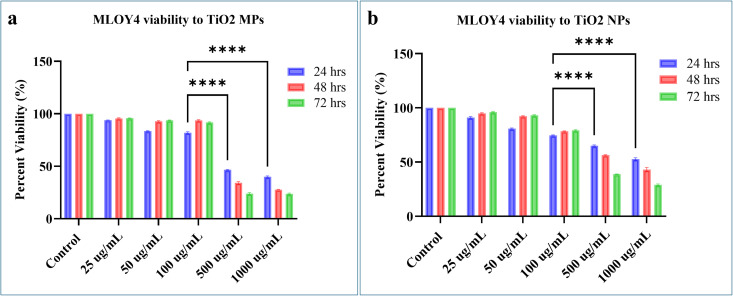
MLO-Y4 cell viability post-treatment with increasing concentrations of TiO2-MPs and NPs. **(a)** Histogram representing MLO-Y4 cell viability post-treatment with 25, 50, 100, 500, 1000 µg/mL of TiO2-MPs at 24, 48, 72 h**. (b)** MLO-Y4 cell viability post treatment with 25, 50, 100, 500, 1000 µg/mL of TiO2-NPs at 24, 48, 72h. All data represent the mean ±SEM of three independent biological experiments, each with three technical replicates. ****p < 0.0001 is indicated.

For MLO-Y4 cells treated with TiO₂-MPs, the percentage viability was considerable up to a concentration of 100 µg/mL with 82% viability at 24 hours. A significant reduction in viability with TiO2-MPs was recorded at higher concentrations from 100 µg/ml onwards, with 500 µg/mL showing 46% viability and 1000 µg/mL showing 40% viability (p < 0.0001; Fig 2a), further declining gradually over time. A similar trend was observed with MLO-Y4 cells treated with TiO₂-NPs (Fig 2b), whereby percentage viability was considerable up to a concentration of 100 µg/mL with 75% viability at 24 hours, while at 500 µg/mL and 1000 µg/mL, cell viability reduced significantly to 65% and 53% (p < 0.0001), respectively.

At lower concentrations of 100 µg/mL and below, both treatment with TiO₂-MPs and NPs exhibited a time-dependent increase in cell proliferation (Fig 2b), whereas concentrations at 500 µg/mL and 1000 µg/mL displayed a time-dependent decrease in cell viability. This concentration maintained the percent cell viability above 50% for both TiO₂-MPs and NPs, even at extended time points.

Based on these findings, 100 µg/ml of TiO₂-MPs and NPs demonstrated a favorable concentration for cell viability under the given experimental conditions and hence selected as the experimental concentration for further studies investigating the potential biological effects of TiO2 particles without causing excessive cytotoxicity.

### 3.2. Time-dependent increase in sclerostin release from MLO-Y4 osteocytes exposed to titanium particles

**Fig 3:**
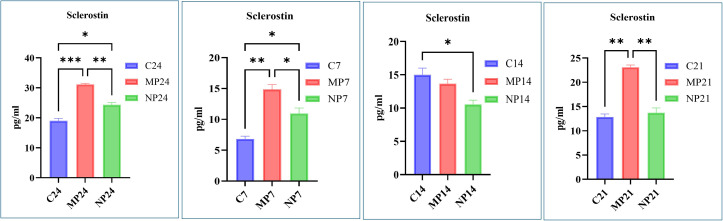
Sclerostin released in the conditioned media of MLO-Y4 osteocytic cells. Bar graphs represent sclerostin release from MLO-Y4 cells treated with 100 µg/mL TiO₂-MPs and NPs along-with untreated control cells at 24 hours, days 7, 14, and 21. All data represent the mean ±SEM of three independent biological experiments, each with three technical replicates. *p < 0.05, **p < 0.01, ***p < 0.001 are indicated.

In the control group, sclerostin levels at 24 hours, days 7, 14, and 21 were 18.99, 6.82, 15.01, and 12.84 pg/ml respectively. In the TiO2-MPs group, the sclerostin levels at these same time points were 31.13, 14.86, 13.7, and 23.06 pg/ml respectively, while for the TiO2-NPs group, the sclerostin levels were 24.3, 10.94, 10.55, and 13.71 pg/ml respectively. Overall, MPs induced a more pronounced and sustained release of sclerostin compared to NPs, with both showing peak expression at 24h hours before gradually declining, suggesting a particle size-dependent and time-dependent cellular responses of TiO2-MPs and NPs (Fig 3).

### 3.3 Alkaline phosphatase expression, RANKL and OPG assays in osteocyte-osteoblast indirect co-culture

**Fig 4:**
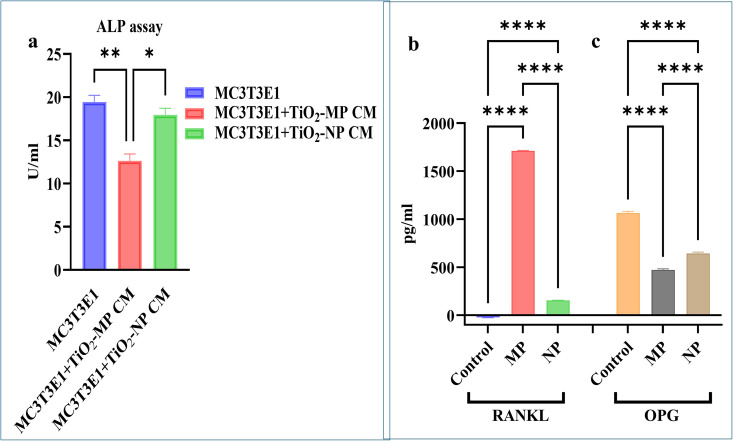
Alkaline phosphatase (ALP) expression, RANKL and OPG release in osteoblasts treated with osteocyte conditioned media (CM) post-treatment with 100 μg/mL TiO2-MPs and NPs. Bar charts representing (a) colorimetric quantification of ALP (U/mL) at day 14 of culture and (b) RANKL and (c) OPG ELISA assays at day 21 of culture. All data represent the mean ±SEM of three independent biological experiments, each with three technical replicates. *p < 0.05 and ****p < 0.0001 are indicated.

ALP assay revealed differences in ALP activity among the indirect osteocyte-osteoblast co-culture groups exposed to MPs CM and NPs CM and the control. We observed a significant decline in ALP activity in MC3T3-E1 cells exposed to MPs CM for 14 days compared to the control MC3T3E1 cells (p < 0.01). Interestingly, cells exposed to NPs CM showed a significant increase in ALP activity compared to MPs CM (p < 0.05) (Fig 4a).

Likewise, the ELISA assay for RANKL and OPG release from osteocyte-osteoblast indirect co-culture exposed to MPs and NPs CM revealed significant differences among the study groups. RANKL release was significantly higher in both MPs CM and NPs CM-treated cells compared to untreated control group (p < 0.0001). When the MPs CM group was compared to NPs CM group, RANKL release was significantly higher in the MPs CM treated group (p < 0.0001; Fig 4b). OPG release was significantly lower in both MPs CM and NPs CM-treated osteoblast cells compared to untreated control group (p < 0.0001). However, when the MP CM group was compared to NP CM, OPG levels were significantly higher in NP CM treated group (p < 0.0001; Fig 4c).

### 3.4. Extracellular matrix mineralization in Osteocyte-Osteoblast indirect co-culture

**Fig 5:**
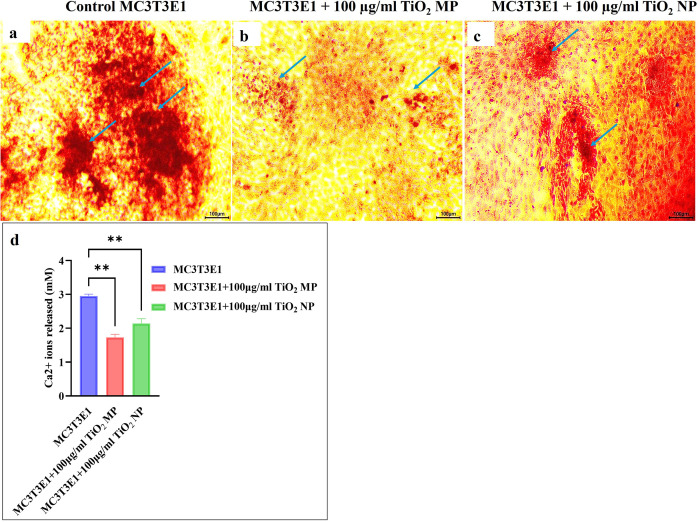
Extracellular matrix mineralization in MC3T3-E1 osteoblast cells after treatment with 100 μg/mL TiO2-MPs CM, and NPs CM obtained from MLO-Y4 osteocyte cells. Microscopic images showing calcification nodules (blue arrows) in (a) untreated control, (b) MPs CM, and (c) NPs CM groups. Scale bar 100 µm for all panels. (d) Quantification of alizarin Red S-stained mineralized nodules showing calcium release at day 21 in culture. All data represent the mean ±SEM of three independent biological experiments, each with three technical replicates. Scale bar = 100 µm for all images. **p < 0.01 is indicated.

Alizarin Red S staining of osteoblast cells revealed notable differences in calcium nodule formation among the untreated control group and MPs CM and NPs CM treated groups, highlighting their differences in osteogenic potential. At 21 days, the amount of calcium deposition was higher in the untreated control group than MPs CM and -NPs CM groups (Fig 5a, b and c). This observation was further supported by the quantification data for Ca2+ ion release, which showed a significant decline (p < 0.01) in both MPs CM and NPs CM groups compared to the control (Fig 5d). Although NPs CM group showed an increase in Ca2+ ion release compared to MPs CM group, the data were not significant.

### 3.5. Gene Expressions for proinflammatory cytokines and bone formation markers in osteocyte-osteoblast indirect co-culture

**Fig 6:**
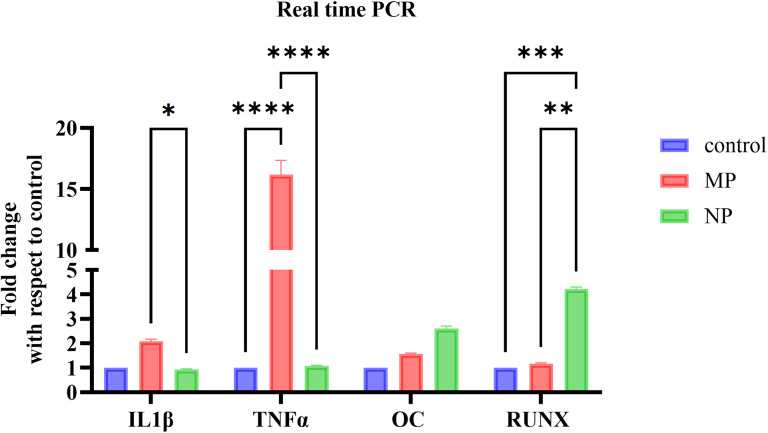
Real-time PCR analysis for expression of proinflammatory cytokines and bone formation markers in MC3T3-E1 cells treated with osteocyte conditioned media (CM) obtained from MLO-Y4 osteocyte cells treated with 100 μg/mL TiO2-MPs and NPs for 21 days. (a) Histograms representing relative changes in mRNA expression of inflammatory cytokines, IL-1β, and TNF-α and bone formation markers like osteocalcin (OC), and Runt-related transcription factor (RUNX2). All data represent the mean ±SEM of three independent biological experiments, each with three technical replicates. *p < 0.05, **p < 0.01, ****p < 0.0001 are indicated.

Exposure of MC3T3-E1 osteoblast cells to MPs CM and NPs CM led to an increase in the mRNA expression of pro-inflammatory cytokines like IL-1β and TNF-α compared to the untreated control. Notably, MPs CM treatment significantly upregulated IL-1β (p < 0.05) mRNA levels at a much higher level compared to the NPs CM. Likewise, MPs CM treatment significantly upregulated TNF-α mRNA levels compared to both the control and NPs CM treatments (p < 0.0001) (Fig 6).

With regards to bone formation markers, exposure of MC3T3-E1 cells to MPs CM and NPs CM led to upregulation of mRNA of OC and RUNX2 osteogenic markers compared to the control, while a significant increase in RUNX2 (*p* < 0.01) was observed in cells exposed to NPs CM compared to MPs CM.

### 3.6. Osteoclast Formation Induced by Osteocyte-MPs and Osteocyte-NPs CM

**Fig 7:**
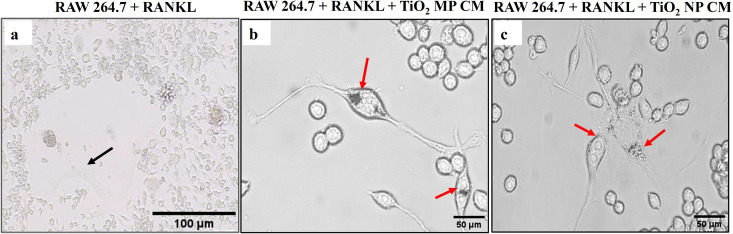
Osteoclast formation by RAW 264.7 cells treated with 100 ng/ml RANKL, with and without exposure to MPs CM and NPs CM for 7 days. **(a)** control: RAW 264.7 cells + 100 ng/ml RANKL **(b)** RAW 264.7 cells treated with 100 ng/ml RANKL and exposed to 100 μg/mL TiO2-MPs CM, and **(c)** RAW 264.7 cells treated with 100 ng/ml RANKL and exposed to 100 μg/mL TiO2-NPs CM. Scale bars = 100 µm and 50 µm are shown. Black arrows depict multinucleated osteoclast cells. Red arrows depict cellular uptake of TiO2-MPs and NPs.

Raw 264.7-derived osteoclasts were observed after 7 days of treatment with 100 ng/mL of murine-derived RANKL. In control RAW 264.7 cells, multinucleated (3 or more nuclei) osteoclasts appeared bigger in size (Fig 7a), while the cells treated with MPs CM (Fig 7b) and NPs CM (Fig 7c) appeared smaller in size compared to the control. Cellular uptake of TiO₂-MPs and NPs was evident microscopically as shown in Figs 7b and c.

### 3.7 Expression of osteoclast markers in osteocyte-osteoclast indirect co-culture

**Fig 8:**
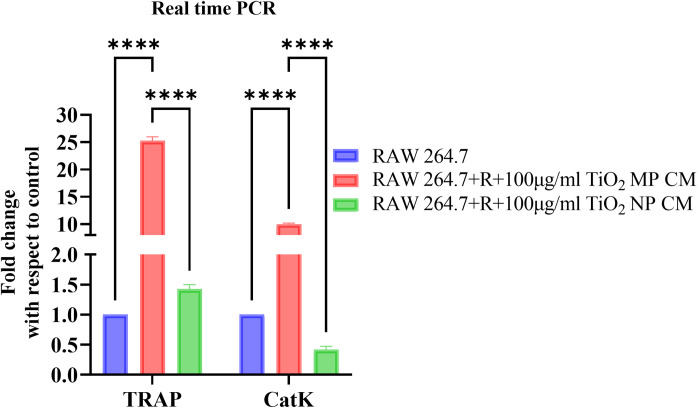
Realtime PCR analysis for expression of osteoclast bone resorption markers by RAW 264.7 cells treated with MPs CM and NPs CM, obtained from MLO-Y4 osteocyte cells treated with 100 μg/mL TiO2-MPs CM (MP CM) and TiO2-NPs CM (NP CM) for 6 days. (a) Histograms representing relative changes in mRNA expression of osteoclastogenic markers TRAP and *Cathepsin-K (CatK)*. All data represent the mean ±SEM of three independent biological experiments, each with three technical replicates. ****p < 0.0001 is indicated.

RAW 264.7 macrophage cells under RANKL stimulation exposed to MPs CM and NPs CM demonstrated a significant increase (p < 0.0001) in the mRNA expression of osteoclastogenic bone resorption markers such as TRAP and *CatK* compared to the untreated control. Notably, MPs CM treatment significantly upregulated TRAP and *CatK* (p < 0.0001) mRNA levels compared to the NPs CM (Fig 8).

## Discussion

While the impact of titanium dioxide particles on immune cells and key bone remodeling cells, including osteoblasts and osteoclasts, is well documented [20,21], a growing body of evidence is now pointing towards the direct role of osteocytes in disrupting bone regenerative balance in particle-driven aseptic osteolysis and loosening of implants. Many interventional studies in this field have focused on inhibitors of osteoclasts and promoters of osteoblasts in attempts to mitigate the harmful effects of titanium dioxide particles, while the influence of these particles on osteocytes has been less investigated [17].

In this study, we have observed significant differences in osteocyte cytotoxicity between TiO₂-MPs and NPs, which may stem from their distinct physicochemical properties. NPs generally have a larger surface area-to-volume ratio and higher reactivity [22], which could influence cellular internalization and interactions with the cell membrane. However, in this study, MPs exhibited a more pronounced effect on osteocyte viability, plausibly due to differences in cellular uptake dynamics or phagocytic burden at equivalent mass concentrations. These factors may affect osteocytic stress responses, particularly on the level and timing of sclerostin secretion.

Osteocytes, being mechano-sensory cells embedded within the mineralized bone matrix, are known to secrete sclerostin upon mechanical unloading [23], which subsequently affects cells in the bone marrow niche via paracrine mechanisms and distant organs through endocrine signaling. The direct effects of titanium particles on SOST gene expression in osteocytes have not been well documented. In this study, treatment of MLO-Y4 osteocytes with TiO2-MPs and NPs led to release of sclerostin in a size and time-dependent manner, in conditions where cell viability remained an acceptable concentration (100 µg/mL). This observation suggests that sclerostin release is not merely a byproduct of cytotoxicity. Rather, it may represent a direct response to particle internalization and subsequent activation of osteocytic signaling pathways, possibly involving inflammatory or Wnt-related pathways [24].

Previous studies have linked osteocyte dysfunction directly to the pathophysiology of several disorders affecting the skeleton [23]. This includes elevated sclerostin and RANKL expression that impairs osteogenic function and increases bone resorption [25]. These signaling shifts disrupt the balance between osteoblast and osteoclast activity, impairing bone matrix deposition and compromising osseointegration. The findings of the current study reinforce such mechanisms, particularly in the context of titanium particle-driven modulation of osteocyte secretions.

Indirect co-culture of CM from TiO₂-MPs or NPs treatment with MC3T3-E1 osteoblasts revealed a reduction in ALP expression compared to control, indicating a lesser osteoblastic differentiation, while RANKL levels were increased, suggesting a pro-osteoclastogenic environment. While osteocytes are the major producers of sclerostin, the protein product of the SOST gene, which antagonizes the canonical Wnt signaling pathway driving osteoblast differentiation [26], they also serve as a dominant source RANKL [27], the key signaling molecule for osteoclast precursor differentiation. Kramer et al. [28] further demonstrated that inactivation of the Wnt/βcatenin pathway in mature osteoblasts and osteocytes downregulates OPG and promotes RANKL expression, facilitating bone resorption. This mechanism may explain why the TiO2-MPs CM group exhibited a much higher pro-osteoclastogenic trend than TiO2-NPs group.

The cross talk between pro-inflammatory cytokines and osteogenic signaling is widely reported [29]. TNF-α and IL-1β, both elevated in the TiO₂-MPs group, are known to suppress osteoblastic differentiation through inhibition of RUNX2 activity and disruption of Wnt signaling. In this study, elevated sclerostin levels in MLO-Y4 osteocytes were accompanied by a high expression of IL-1β and TNF-α in the osteocyte-osteoblast co-culture, indicating an intense pro-inflammatory condition. TNF-α not only supports osteoclastogenesis but has also been reported to upregulate sclerostin expression in osteocytes by both *in-vitro* and *in-vivo* studies [30]. Similarly, IL-1β plays a major role in bone repair and remodeling and has recently been shown to promote the expression and secretion of sclerostin in osteocyte-osteoblast co-culture models [31]. The higher pro-inflammatory cytokine profile in the TiO₂-MPs group supports the idea that particle size indirectly influences sclerostin release via cytokine-mediated signaling.

Extracellular matrix mineralization, the final phenotypic marker of osteoblasts differentiation, was significantly impaired in co-cultures with TiO₂-treated osteocyte CM. In this study, by day 21, the osteoblasts alone control group showed a high amount of mineralization nodules within the extracellular matrix, while the osteocyte-osteoblast co-culture group, particularly with TiO₂-MPs CM, demonstrated reduced nodule formation, as indicated by Alizarin Red staining. This reduction likely resulted from sclerostin release from osteocytes, which may have played a critical role in disrupting mineralized nodule formation by inhibiting osteoblast activity, thus affecting the mineralization of the extracellular matrix. In this study, the inhibition of Wnt/β-catenin signaling pathway by sclerostin may have downregulated the expression of genes involved in bone formation and mineralization, reducing the expression of mineralization proteins such as alkaline phosphatase (ALP) that is critical for hydrolyzing pyrophosphate (an inhibitor of mineralization) and promoting the deposition of calcium and phosphate ions in the ECM [32]. Additionally, sclerostin-enhanced RANKL expression and suppression of OPG likely shifted the remodeling balance toward bone resorption [33]. The co-culture group with CM involving TiO2-MPs demonstrated considerably less amount of mineralization nodules in the extracellular matrix compared to those treated with TiO2-NPs, suggesting micro-size particles exposed to osteocytes are more harmful than nanoparticles since it resulted in less osteogenic potential as found in this study. This observation is further supported by the significantly elevated levels of RANKL and decreased levels of OPG released in the TiO2-MPs CM group when compared to both the control and TiO2-NPs CM groups.

Expression of bone formation markers such as OC and RUNX2 were increased in the co-culture groups compared to control, with the NPs CM group showing significantly higher RUNX2 expression than the MPs group. We hypothesize the reason for these findings could be due to the biofeedback mechanism of this complex regulatory system, helping to maintain bone homeostasis during remodeling [34,35].

Further investigations on the influence of indirect co-culture of TiO2treated osteocyte with osteoclasts were performed by treating MLO-Y4 with TiO2-MPs and NPs and exposing the CM to RAW 264.7 cells. In the osteoclast formation assay, by day 7, the presence of multi-nucleated osteoclast-like cells, and gene expression of TRAP and *Cat K* were significantly higher in theTiO2-MPs group than the control, indicating successful osteoclast differentiation. While this study did not include viability staining in osteoclast precursors, the robust gene expression and morphology suggest that RANKL and sclerostin in the conditioned media may have promoted osteoclastogenesis by enhancing RANKL signaling and suppressing OPG. These findings align with the observations by Asiri R Wijenayaka et al., who reported that sclerostin enhances osteocyte-mediated osteoclast support via a RANKL-dependent mechanism [36]. The insights obtained from the current study support the development of osteocyte-secretome targeted therapies as a potential strategy for mitigating failed implants.

## Conclusions

The osteocytes are now recognized as a major orchestrator of skeletal activity, capable of sensing and integrating mechanical and chemical signals from their environment to regulate both bone formation and resorption. The novelty of this study is highlighted through elucidating the mechanistic role of osteocytes in titanium implant wear particle–induced bone remodeling. We demonstrate that titanium dioxide microparticles stimulate osteocytes to modulate the local microenvironment through altered sclerostin and RANKL signaling, suppressing osteoblastogenesis and enhancing osteoclastogenesis. Sclerostin demonstrated a direct action on preventing osteoblastogenesis and RANKL promote osteoclastogenesis shifting the homeostatic balance towards bone resorption. By inhibiting osteoblast activity and lowering OPG expression, sclerostin increases the availability of RANKL, which is crucial for driving osteoclastogenesis. This study highlighted the role of osteocytes in aseptic osteolysis following particle-lead disruption in bone remodeling suggesting sclerostin as a possible target in mitigating failed implants.
